# Nutritional Status among the Children of Age Group 5-14 Years in Selected Arsenic Exposed and Non-Exposed Areas of Bangladesh

**Published:** 2014-12

**Authors:** Mohammad Rezaul Karim, Sk. Akhtar Ahmad

**Affiliations:** 1 Department of Population Dynamic, National Institute of Preventive and Social Medicine (NIPSOM), Mohakhali, Dhaka, Bangladesh; 2 Department of Occupational and Environmental Health, Bangladesh Institute of Health Service (BISH), Darussalam, Mirpur, Dhaka, Bangladesh

**Keywords:** Source of water, Arsenic level, Tube well water, Dietary intake, Height, Weight, Stool parasite

## Abstract

**Objective:** To assess and compare the nutritional status of children aged 5-14 years in arsenic exposed and non- exposed areas.

**Materials and methods:** It was a cross sectional study conducted on 600 children of age 5-14 years from arsenic exposed and non-exposed areas in Bangladesh. Designed questionnaire and check list were used for collection of data. To estimate BMI necessary anthropometric measurements of the studied children were done. Dietary intakes of the study children were assessed using 24-hours recall method.

**Results:** The difference of socio-economic conditions between the children of exposed area and non-exposed area was not significant. On an average the body mass index was found to be significantly (p < 0.01) lower among the children of arsenic exposed area (49%) in comparison to that of children in non-exposed area (38%). Stunting (p < 0.01), wasting (p < 0.05) and underweight (p < 0.05) were significantly higher in exposed group in comparison to non-exposed group. No significant difference of nutrition intake was found between exposed and non-exposed children as well as thin and normal children.

**Conclusion:** In this study children exposed to arsenic contaminated water were found to be suffered from lower nutritional status.

## Introduction

The threat to public health by arsenic contamination in drinking water has attracted much attention since the 1990s, largely due to the scale of the problem in Bangladesh which was described as “ the largest poisoning of a population in history” (1). Water is the most abundant resource in Bangladesh, but arsenic concentration of ground water has become a matter of serious concern. It is the most extensive environmental disaster of the twentieth century. The problem of arsenic contamination of ground water in the subcontinent was recognized first in West Bengal, India in 1983 (2-5). Though Bangladesh shares a common border with India and similar geomorphologic features in West Bengal, the possibility of having the same problem in Bangladesh was not anticipated until 1993 when WHO raised the possibility of arsenic contamination in tube well water in areas adjoining West Bengal (6-8). The government of Bangladesh officially recognized the existence of the problem following detection of arsenic contamination in water of four tube wells in the village Chamagram under the district of Nawabgonj by the Department of Public Health Engineering (DPHE) in 1993 (1, 9-11). However, the Department of Occupational and Environmental Health (DOEH), National Institute of Preventive and Social Medicine (NIPSOM) identified 8 patients in 1994 in the same area which were reported to be first identified arsenicosis cases (12,13). Now it has been reported that about 30 million to 50 million people are at risk of arsenic exposure (6,14,15). According to the recent report of Director General of Health Service (DGHS).The arsenic contamination in the tube well water has been detected in 62 out of 64 districts (1, 16, 17). Bangladesh Arsenic Mitigation and Water Supply Project (BAMWSP*)* screened tube wells in 271 Thana out of 490 Thana and arsenic contamination was found in 29.2% of the tube well and so, far, 38,500 arsenicosis patients had been identified (18). It has been estimated that about 29 million people in Bangladesh are exposed to drinking water with arsenic exceeding Bangladesh standard 0.05 mg/L (16,19). A WHO report predicted that in most of the southern part of Bangladesh almost 1 in 10 adult deaths will be a result of cancer triggered by arsenic poisoning in the next decade.

 Chronic arsenic exposure increases the risk of death and infant mortality (20). It has been reported that person taking arsenic contaminated water for 2-10 years develop arsenicosis. Infants and children are considered to be more susceptible to the adverse effects of arsenic exposure (2). The youngest reported arsenicosis patient in Bangladesh was 4 years old (6). Nutrition plays a decisive role in the prevention of the onset of arsenic related ailments. Alternatively it was also reported that arsenic exposure may contribute to poor nutritional status (19- 22). There is evidence that people in poor socio-economic conditions are more prone to develop arsenicosis (21). In Bangladesh almost one fourth of the population is children but the effect of arsenic toxicity particularly effect on nutritional status amongst them not been well documented (23). This study was carried out to explore the nutritional status of the children of arsenic contaminated area, on the basis of which appropriate measures could be under taken for their future health development.

## Materials and methods

This cross sectional comparative study was carried out among the children of arsenic exposed and non-exposed area of Bangladesh. Children of 5-14 years of age were the study population. A total of 910 from arsenic exposed area and 920 from arsenic non-exposed area were included in the study. Amongst the selected study children who were found to be suffered from helminthiasis as evident by their stool examination report, were excluded from the study. From the list of the children who fulfilled the inclusion criteria, a total of 300 children from arsenic exposed area and 300 children from non-exposed area were randomly selected as respondent of the study population. A pre-tested questionnaire and a checklist were used for collection of data. To estimate BMI necessary anthropometric measurements were done for all the study children. Twenty four hours recalled questionnaire was used for dietary assessment of the study population. The village where more than 80% of tube wells are reported to be Arsenic contaminated was considered as arsenic exposed area and where all most all the tube well are not arsenic contaminated was considered as arsenic non-exposed area for this study.

## Results

No significant differences in socio-economic characteristic of children between exposed and non- exposed area were found (Table 1).

The nutrients intake such as protein, fat and carbohydrate and vitamins taken by the children per day of both exposed and non-exposed group had no significant difference (Table 2).

The average height and weight of the exposed children were found to be lower in comparison to that of non-exposed children and the difference was statistically significant (table 3). The mean Body Mass Index (BMI) of the children in the exposed group was ^2^^2 ^in the non-exposed group. Body mass index (BMI) of the non-exposed group children was found to be significantly higher than that of the exposed group (p< 0.05) (Table 3).

The BMI in percentile based in terms of thinness was found more among the exposed children (59.3%) while normal BMI was more among the non-exposed children (68.7%). The differences were statistically significant (p < 0.01) (Table 4).

While comparing the nutrient intake by thinness and normal children it was found that none of the nutrients significantly differ between two groups (Table 5).

**Table 1:** Socio-economic characteristics of the study children

	Study area		Total (n= 600)	P value
	Exposed (n= 300)	Non-exposed (n= 300)		
**Age group of children (years)**				
**Mean± SD**	**8.8± 2.6**	**8.4± 2.4**	**8.6± 2.5**	**0.110**
**Range**	**5- 14**	**5- 14**	**5- 14**	
**Sex of the children Boys and Girls)**				
**Mean± SD**	**8.7± 2.5**	**8.5± 2.5**	**8.6± 2.5**	**0.437**
**Range**	**5.0- 14.0**	**5.0- 13.9**	**5.0- 14.0**	
**Age of the respondents (years)**				
**Mean± SD**	**41.2± 6.9**	**41.6± 6.6**	**41.4± 6.8**	**0.530**
**(Father)**	**29- 70**	**28- 65**	**28- 70**	
**Mean± SD**	**32.5± 5.9**	**32.5± 5.7**	**32.5± 5.8**	**0.922**
**(Mother)**	**22- 55**	**20- 52**	**20- 55**	
**Respondent’ s family size **				
**Mean± SD **	**5.04± 0.9**	**5.11± 1.0**	**5.08± 0.9**	**0.411**
**(Range**	**3- 7**	**3- 8**	**3- 8**	
**Monthly in of the respondents**				
**Mean± SD **	**4015.0± 1246.6**	**3861.7± 1348.7**	**3938.3± 1299.9**	**0.149**
**(Range**	**2000- 7000**	**1500- 15000**	**1500- 15000**	

**Table 2:** Amount of principal nutrients taken per day by the study children

	**Amount taken per day**		
	**Exposed (n = 300)** **(Mean ** **±** ** SD)**	**Non-exposed (n = 300)** **(Mean ** **±** ** SD)**	**Significance**
Total weight (g) of food	714.39 ± 226.98	693.6311 ± 217.88	t =1.143; p= 0.254
Energy (kcal)	1085.91 ± 714.84	1048.72 ± 296.63	t = 0.832; p = 0.406
Protein (g)	33 28	30 24	p =0.212; p = 0.212
Fat (g)	9 8	11 22	P =0.124; p = 0.124
Carbohydrate (g)	331.64 ± 197.34	330.59 ± 198.27	t = 0.065; p = 0.949
Ca (mg)	290.14 ± 249.39	305.00 ± 214.16	t = 0.784; p = 0.434
Iron (mg)	9.1868 ± 6.92	9.27 ± 7.31	t = 0.146; p = 0.884
Ribo (mg)	0.4494 ± 0.28	0.4931 ± 0.66	t = 1.054 ; p = 0.292
Thia (mg)	0.6309 ± 0.18084	0.6303 ± 0.18041	t =.038; p = 0.969
Zinc (gm)	4.36 ± 3.12	4.34 ± 2.51	t = -0.097; p = 0.923
Vitamine A(IU)	347.61 ± 1652.03	375.00 ± 1897.26	t = 0.189; p = 0.850
Vitamine C(mg)	24.39 ± 22.37	23.04 ± 18.23	t = 0.809; p = 0.419
Carotein((ugm)	415.06 ± 624.78	442.59 ± 784.25	t = -0.476; p = 0.635
Niacine(mg)	11.26 ± 8.15	10.60 ± 3.97	t = -1.270; p = 0.205

**Table 3:** Respondents by anthropometric measurements

	**Mean**	**SD**	**Minimum**	**Maximum**	**p value**
Height					
Exposed	119.77	13.32	86.50	151.70	t= 3.527 p< 0.001
Non-exposed	123.81	14.77	86.50	155.50	
Weight					
Exposed	21.19	13.317	90.00	151.70	t= 3.746 p< 0.001
Non-exposed	23.53	14.76	86.50	155.50	
BMI					
Exposed	14.42	2.20	8.58	23.97	t= 2.52 p= 0.012
Non-exposed	14.87	2.16	10.49	24.81	

The analysis of z-score of anthropometric measurement was performed to assess the physical growth of the children in terms of stunting (height for age), wasting (weight for height) and underweight (weight for age) of the children. It was found that among the children of exposed group stunting (57%), wasting (67%) and underweight (68%) were found significantly higher compared to those of non-exposed group (Table 6). 

***Correlates of malnutrition: Binary logistic regression analysis***

To assess the factors influencing the malnutrition among the children binary logistic regression analysis was carried out in which the dependent variable, ‘nutritional status’ was dichotomized (malnourished/ normal). For prediction of influencing factors for malnutrition, variables that showed significant association with nutritional status, in chi-square analysis were entered into logistic regression model. The nutritional status of the children was assessed by z- score of weight for age, height for age and weight for height. Children having any of the parameters in terms of underweight, stunting and wasting were considered as malnutrition cases (Table 7).

**Table 4:** Respondents by BMI in percentile group

	**Exposed (n = 300)**	**Non-exposed (n = 300)**	**p value**
Thinness (Low BMI for Age) n (%)	178 (59.3)	82 (27.3)	p < 0.01
Normal BMI for Age n (%)	114 (38.0)	206 (68.7)	
Overweight (High BMI for Age) n (%)	8 (2.7)	12 (4.0)	

**Table 5:** Amount of principal nutrients taken per day and correlation of BMI with principal nutrient taken by the study children

	**Amount taken per day**		
	**Thinness**	**Normal & Over Weight**	**Significance**
Total food (gram)	705.14 ± 220.83	703.15 ± 224.14	t = 0.109; p = 0.913
Energy (kcal)	1040.50 ± 290.26	1087.82 ± 680.87	t = -1.050 p = 0.294
Protein (gram)	29.91 19.23	32.66 29.95	t = -1.289; p = 0.198
Fat (gram)	9.63 8.71	11 20.76	t = -0.992; p = 0.321
Carbohydrate (gram)	338.93 ± 199.02	325.13 ± 196.66	t =0.847; p = 0.397
Ca (mg)	285.00 ± 250.17	307.18 ± 217.66	t = -1.159; p = 0.247
Iron (mg)	8.81 ± 6.48	9.55 ± 7.55	t = -1.264; p = 0.207
Riboflavin (mg)	0.4326 ± 0.28	0.5009 ± 63348	t = -1.635; p = 0.103
Thiamin (mg)	0.6404 ± 0.26214	0.6231 ± 0.16882	t = 1.168; p = 0.243
Zinc (gm)	4.08 ± 1.48	4.56 ± 3.52	t = -2.021; p = 0.044
Vitamine A (IU)	272.24 ± 646.96	429.42 ± 291.69	t = -1.074; p = 0.283
Vitamine C (mg)	22.61 ± 18.82	24.56 ± 21.52	t = -1.158; p = 0.247
Carotein (µgm)	445.68 ± 743.73	415.94 ± 681.26	t = 0.509; p = 0.611
Niacine (mg)	10.35 ± 5.54	11.37 ± 6.98	t = -1.941; p = 0.053

Out of 8 variables, 5 variables showed significant association in binary logistic regression analysis. The analysis showed that subjects exposed to arsenic contamination, duration of tube well water use, frequency of taken per week and number of glass of water drink per day appeared to be the main prediction of malnutrition among the children p< 0.001. Data analysis indicated that the malnutrition was found to be 4.2 times higher among the children who consumed wet rice more than 2 times per week, 7.2 times higher among the children exposed to arsenic in water. In the logistic model, showed that the malnutrition of exposed group was significantly positively correlated with frequency of pulses, wet rice and number of glass of water drinks per day indicating the children were more exposed to arsenic contamination through pulses, wet rice and also arsenic contaminated water.

**Table 6:** Children by categorization of nutritional status according to z-score

	**Exposed** **n (%)**		**Non Exposed** **n (%)**	**Total** **n (%)**			**p value**
Height for age							
Normal	199 (66.3)	224 (74.7)			423 (70.5)	0.025	
Stunting	101(33.7)	76 (25.3)			177 (29.5)		
Weight for age							
Normal	264 (88.0)	283 (94.3)			547 (91.2)	0.006	
Underweight	36 (12.0)	17 (5.7)			53 (8.8)		
Weight for height							
Normal	277 (92.3)	289 (96.3)			566 (94.3)	0.034	
Wasting	23 (7.7)	11 (3.7)			34 (5.7)		

**Table 7:** Correlates of malnutrition among the children: Binary logistic regression analysis

**Independent variables **	**β**	**df**	**p value**	**Odds ratio**	**95.0% C.I**
Subject					
Non exposed RC	-	-	-	-	-
Exposed	1.978	1	0.000	7.230	2.915- 17.931
Sources of water					
Tube well RC	-	-	-	-	-
Others sources	0.305	1	0.471	1.357	0.592- 3.111
Age in years Father					
<40 RC	-	-	-	-	-
≥40	-0.845	1	0.053	0.429	0.183- 1.010
Age in years Mother					
<30 RC	-	-	-	-	-
≥30	-0.213	1	0.648	0.808	0.324- 2.015
Duration of Tube well water use yrs					
<7 RC	-	-	-	-	-
≥7	1.263	1	0.000	3.537	1.771- 7.063
No. of wet rice taken per week by children					
<2					
≥2	1.449	1	0.000	4.261	2.122- 8.556
Frequency of pulses taken per week	1.432	1	0.000	4.186	3.207- 5.464
No. of glass of water taken per day	.626	1	0.000	1.869	1.596- 2.189
Model Chi square	498.898	5	0.001		
df	5				
Significance	0.001				
Constant	-12.145				

RC = Reference category, CI = Confidence interval

## Conclusion

This cross sectional study was designed to compare the nutritional status of 5-14 years age children of selected arsenic non-exposed and arsenic exposed areas. Probable determinants (socio-demographic characteristics, principal dietary intake etc) were considered to assess any relation with nutritional status. The study findings suggest that there was no remarkable difference in overall socio-economic status (e.g. income, education, occupation etc.) between exposed and non-exposed areas. Dietary consumption also did not show any gross difference between these two groups. However, the study revealed significantly lower number of underweight children in the non- exposed area in comparison to exposed area. It was found that the lower number of underweight in non-exposed area was significantly influenced (p < 0.05) by older age group 11-14 years of children. Chi square tests were performed to see the associations between exposure and effect with confounder’s: monthly income and family size. dal and number of glass of water drinks per day indicating the children were more exposed to arsenic contamination through foods and also water. So, the lower nutritional status observed among the arsenic exposed children compared to non-exposed children in this study seems to be attribute to arsenic exposure. 

## Confilict of Interests

Authors declare that they have no conflict of interest.
